# The effects of casticin and myricetin on liver damage induced by methotrexate in rats

**DOI:** 10.22038/ijbms.2018.29922.7217

**Published:** 2018-12

**Authors:** Fazile Nur Eki̇nci̇-Akdemi̇r, Serkan Yildirim, Fatih Mehmet Kandemi̇r, İlhami Gülçi̇n, Sefa Küçükler, Yavuz Selim Sağlam, Selvinaz Yakan

**Affiliations:** 1 Department of Nutrition and Dietetics, Health School, Ağrı İbrahim Çeçen University, Ağrı, Turkey; 2 Department of Pathology, Faculty of Veterinary, Atatürk University, Erzurum, Turkey; 3 Department of Biochemistry, Faculty of Veterinary, Atatürk University, Erzurum, Turkey; 4 Department of Chemistry, Faculty of Science, Atatürk University, Erzurum, Turkey; 5 Department of Animal Health, School of Eleşkirt Celal Oruç, Ağrı İbrahim Çeçen University, Ağrı, Turkey

**Keywords:** Antioxidants, Casticin, Myricetin, Oxidative stress, Reactive oxygen species

## Abstract

**Objective(s):**

In this study, we evaluated the therapeutic effects of casticin and myricetin on liver damage induced by methotrexate in rats.

**Materials and Methods:**

Thirty-six male rats were used for the study and divided into 6 groups: control, methotrexate, casticin, myricetin, casticin+methotrexate, and myricetin+methotrexate. It was performed by methotrexate (20 mg/kg single dose, IP) in methotrexate, casticin+methotrexate and myricetin+methotrexate groups. Casticin 200 mg/kg dose was given to casticin and casticin+methotrexate groups. Myricetin 50 mg/kg dose was given to myricetin and myriceytin+methotrexate groups. At the end of the experiment, liver tissues were removed for the purpose of histopathological, biochemical and immunohistochemical assessments.

**Results:**

In our study, we have detected that MDA levels increased and activities of antioxidant enzymes SOD, CAT, and GPX decreased in the methotrexate group compared to the other groups, but the level of MDA decreased and activities of these enzymes increased in casticin+methotrexate and myricetin+methotrexate groups compared to the methotrexate group. In immunohistochemical examinations of control, casticin and myricetin groups in liver tissues no caspase-3 and 8-OHdG expressions were observed. In the MTX group, caspase-3 and 8-OHdG expressions were seen at the severe levels. Caspase-3 and 8-OHdG expressions were mild in hepatocytes in the casticin+methotrexate and myricetin+methotrexate groups. When the liver tissues of the rats in the methotrexate group were examined, severe pathological damage was detected both in the parietal region and in the portal region.

**Conclusion:**

By looking at these results, we can say that casticin and myricetin are effective against liver damage induced by methotrexate.

## Introduction

Methotrexate (MTX), a folic acid antimetabolite, is used as antineoplastic drug acting in the treatment of many conditions such as various cancers, rheumatoid arthritis and psoriatic arthritis (1, 2). Methotrexate has many application fields as a therapeutic agent at high doses in many malignancies and at low doses in autoimmune diseases (3). Despite these indications of wide usage, MTX has drawn attention with a range of side effects such as nephrotoxicity and hepatotoxicity (4, 5). The main mechanism of MTX-mediated liver toxicity has not yet been clarified fully. However, there are a number of scientific studies on the hypothesis that oxidative stress results in a large amount of free radical formation as one of the main reasons. Therefore, it has been noticed that MTX causes the oxidative injury of the DNA and triggers lipid peroxidation. Overproduction of reactive oxygen species (ROS) implicated in MTX hepatotoxicity depletes cellular enzymatic and non-enzymatic antioxidant defense systems (6, 7). Recent research results reported that MTX treatment can start a reduction in NADPH, GSH, increase the formation of ROS, and hepatotoxic determiners connected with decreased or inadequate cellular antioxidant defense system, resulting in hepatic oxidative damage (8). The preference of various non-toxic cytoprotective agents as supportive may play a significant role to attenuate the intensity of undesirable effects of MTX chemotherapy with protection of chemotherapeutic efficacy. Tissue and organ protection against toxicity caused by agents with chemotherapeutic effect is accepted to be a very important goal and the objective of many studies on supporting antioxidant treatment (9, 10). The usage of natural compounds or drugs, non-toxic cytoprotective agents as adjuvants, may play a significant role to decrease the incidence of side effects of MTX chemotherapy with preservation of chemotherapeutic efficacy.

Numerous research studies to date have reported that many natural compounds have protective activity in the liver tissue. It has been shown that casticin (3′, 5-dihydroxy-3, 4′, 6, 7-tetramethoxyflavone) is one of the major flavonoids in *Fructus viticis* extracts often used in traditional treatment methods for various pains such as migraine, chronic headaches, and eye pain (11). It has been reported that casticin (CAS) shows anti-oxidant and anti-inflammatory effects and anticancer activities (12, 13). Myricetin (MYR) is a bioactive flavonol, an effective antioxidant, abundant in various fruit derivatives such as peanuts, strawberries, and grapes. MYR has been proven to have anti-cancer, antiproliferative, anti-angiogenic, and anti-invasive effects on many types of cancer, including ovarian, stomach, and colon cancers (14, 15).

MTX is the indispensable drug for some cancer patients. However, the side effects can lead to dose restriction or termination of treatment, which reduces the patient’s survival. It is very important clinically to remove the side effects without causing it. Therefore, we aimed to see the protective effects of CAS and MYR against hepatotoxicity induced by MTX. For this purpose we administered a single dose of MTX (20 mg/kg, IP) and examined the effects of CAS and MYR on the histopathological changes and levels of caspase-3 and 8-OHdG expressions and MDA level, which is an important indicator of lipid peroxidation, and activities of antioxidant enzymes such as superoxide dismutase (SOD), catalase (CAT), and glutathione peroxidase (GPX) in the liver tissue of rats.

## Materials and Methods

***Drugs***

MTX (50 mg/ 5 ml injectable solution) was provided by Koçak Farma (Istanbul, Turkey). CAS and MYR were obtained from Sigma-Aldrich Chemical Company, USA. The dose of MTX was chosen based on a recent study reported by Arslan (16). CAS and MYR were dissolved in DMSO. Dosages of CAS (17) and MYR (18) were selected from those described by Ling *et. al.* and Sun *et. al*., respectively. Caspase 8 and 8-OHdG antibody rat kits were purchased from Sigma-Aldrich Chemical Company, USA.

***Ethical approval and experimental design***

This study has been approved by the Animal Experiment Local Ethics Committee of Atatürk University (date: 2017, no: 146). The rats were maintained under pathogen-free conditions with air conditioning, a 12-hr light/12-hr dark cycle, and %55 humidity. In addition, all of the rats had free access to food and water during the experiments. In our study, we used 36 Wistar type male rats (age; 9 weeks, weight; 210-230 g). Rats were weighed and 6 experimental groups were formed.

1. Control group; no treatment was applied in this group.

2. MTX group; on the first day of the experiment, a single MTX dose (20 mg/kg, IP) was administered.

3. MYR group; MYR (50 mg/kg, IP) was administered for 5 days to this group.

4. CAS group; CAS (200 mg/kg, IP) was administered for 5 days to this group.

5. MYR+MTX group; After administration of methotrexate as described in group 2, MYR (50 mg/kg, IP) treatment continued for 5 days in this group.

6. CAS+MTX; after administration of MTX as described in the MTX group, CAS (200 mg/kg, IP) treatment continued for 5 days in this group.

At the end of the experiments, all rats were sacrificed under high-dose anesthesia. The liver tissues were precisely removed without loss of time, washed with cold saline, and part of liver tissue preserved at -80 ^°^C for biochemical analysis. The rest of the liver tissue was kept in 10% formalin for pathological and immunohistochemical analysis.

***Determination of lipid peroxidation and antioxidant enzymes analysis***

The malondialdehyde (MDA) (nmol/g tissue) level was determined according to the method by Placer *et al.* (19). Superoxide dismutase (SOD) activity was measured by the method of Sun *et al.* (1988) (20). The catalase (CAT) (katal/g protein) activity was assessed by the method of Aebi (1983) (21). Glutathione (GSH) (nmol/g tissue) was assessed by the method of Sedlak and Lindsay (1968) (22). The glutathione peroxidase (GPx) (U/g protein) activity was calculated by the method of Lawrence and Burk (1976) (23). The protein content of the supernatant was determined using the Lowry method (24).

***Histopathological examination***

Tissue specimens of the liver taken for histopathological evaluations in results of necropsy were detected in 10% formalin solution for 48 hr. As routine tissue tracking procedures, samples were embedded in paraffin blocks. Sections of 4 µm thickness were taken from each block. Preparations prepared for histopathological examination were stained with hematoxylin-eosin (HE) and examined by light microscopy. (Leica DM 1000, Germany). The sections were evaluated as no (-), mild (+), moderate (++), and severe (+++) according to histopathologic findings.

***Immunohistochemical examination***

For immunoperoxidase examination, all sections taken with adhesive (poly-L-Lysine) slides were sacrificed and dehydrated by passing through xylol and alcohol series. Then they were washed in distilled water for 5 min. The sections were microwaved 4 times for 5 min in an antigen retrieval (citrate buffer, pH 6.1) solution to prevent masking of the antigen in the core, then removed from the microwave oven and allowed to cool to room temperature for 30 min. At the end of this duration, sections were washed with distilled water, dried around the sections and drawn with special glass pencil. Endogenous peroxidase was inactivated by keeping in 3% H_2_O_2_ for 10 min and washing with phosphate buffered solution (PBS, pH 7.2) for 5 min. After washing in PBS for 5-10 min, they were incubated for 5 min with protein block compatible with all primer and secondary antibody to prevent nonspecific ground staining. At the end of the incubation, the primary antibody (caspase-3, 8-OHdG, and PBS in the control group) was distilled without washing the block solution remaining on the tissue sections. In accordance with the primer, the antibody was allowed to stand at room temperature for 1 hr. Washed twice with PBS for 5 min each and incubated with biotinised secondary antibody for 10-30 min at room temperature. Sections were washed again with PBS after keeping in streptavidin-peroxidase for 10-30 min, washed in the same manner as PBS. For floor coating in Mayer’s hematoxylin, they were kept for 1-2 min and then washed in tap water. After this process, lams were closed by keeping in 80% ethanol, 96% ethanol, 100% ethanol, and xylolite for 3 min the lamellae were closed with the help of the intramolecular blades. The sections were evaluated as no (-), mild (+), moderate (++) and severe (+++) according to their immunity positivity.

***Statistical Analysis***

Biochemical data comparisons were performed using analysis of variance (ANOVA) followed by Tukey’s multiple comparisons test for *post hoc* analysis. Histopathological findings were analyzed with nonparametric Kruskal-Wallis test and Mann-Whitney U test for the comparison of the binary groups for the analysis of the differences between the groups semiquantitatively in histopathological examinations. 

## Results

***Biochemical results***

**Figure 1:**
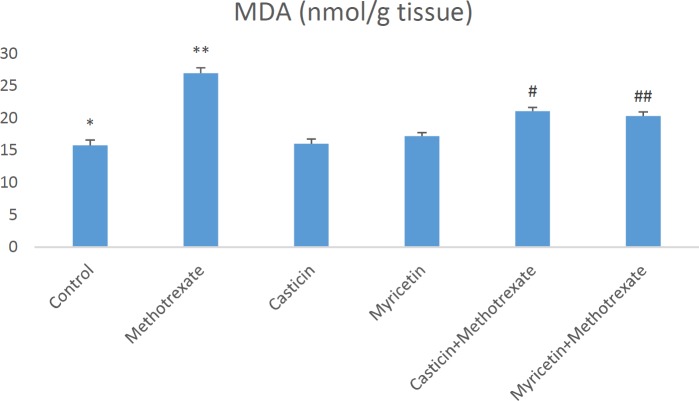
A significant difference between control and methotrexate, CAS+methotrexate, MYR+methotrexate groups (*P<*0.05). **: A significant difference between methotrexate and control, CAS, MYR, CAS+methotrexate, and MYR+methotrexate groups (*P<*0.05). #: A significant difference between CAS+methotrexate and control, methotrexate, CAS, and MYR groups (*P<*0.05). ##: A significant difference between MYR+methotrexate and control, methotrexate, and CAS groups (*P<*0.05)

**Figure 2:**
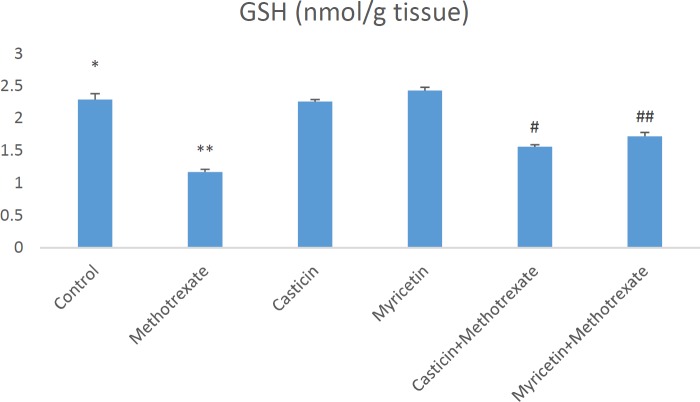
A significant difference between control and methotrexate, CAS+methotrexate, MYR+methotrexate groups (*P<*0.05). **: A significant difference between methotrexate and control, CAS, MYR, CAS+methotrexate, and MYR+methotrexate groups (*P<*0.05). #: A significant difference between CAS+methotrexate and control, methotrexate, CAS, and MYR groups (*P<*0.05). ##: A significant difference between MYR+methotrexate and control, methotrexate, CAS, and MYR groups (*P<*0.05)

**Figure 3.**
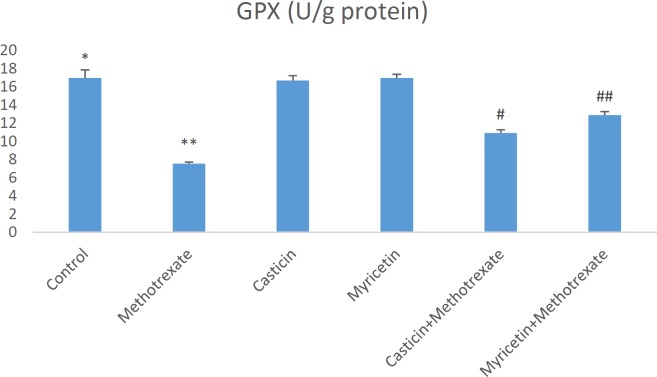
A significant difference between control and methotrexate, CAS+methotrexate, and MYR+methotrexate groups (*P<*0.05). **: A significant difference between methotrexate and control, CAS, MYR, CAS+methotrexate, and MYR+methotrexate groups (*P<*0.05). #: A significant difference between CAS+methotrexate and control, methotrexate, CAS, MYR, and MYR+methotrexate groups (*P<*0.05). ##: A significant difference between MYR+methotrexate and control, methotrexate, MYR, CAS, and CAS+methotrexate groups (*P<*0.05)

**Figure 4:**
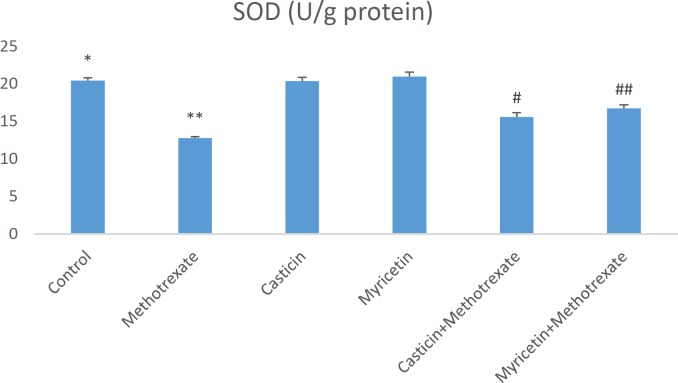
A significant difference between control and methotrexate, CAS+methotrexate, and MYR+methotrexate groups (*P<*0.05). **: A significant difference between methotrexate and control, CAS, MYR, CAS+methotrexate, and MYR+methotrexate groups (*P<*0.05). #: A significant difference between CAS+methotrexate and control, methotrexate, CAS, and MYR groups (*P<*0.05).##: A significant difference between MYR+methotrexate and control, methotrexate, CAS, and MYR groups (*P<*0.05)

**Figure 5:**
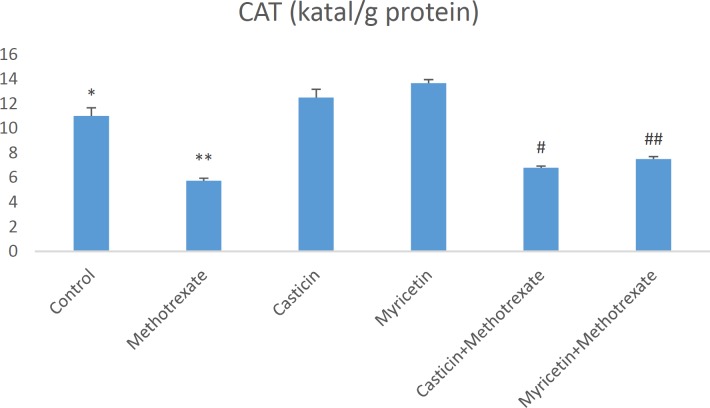
A significant difference between control and methotrexate, CAS+ methotrexate, and MYR+methotrexate groups (*P<*0.05). **: A significant difference between methotrexate and control, CAS, MYR, CAS+methotrexate, and MYR+methotrexate groups (*P<*0.05). #: A significant difference between CAS+ methotrexate and control, methotrexate, CAS, and MYR groups (*P<*0.05). ##: A significant difference between MYR+methotrexate and control, methotrexate, MYR, and CAS groups (*P<*0.05)

Biochemical parameters in liver damage caused by MTX are presented as mean±SEM in all groups. When MDA (nmol/g tissue) results were evaluated, a significant increase in MDA formation was observed in the MTX group compared to the control group. However, MDA levels decreased in CAS+MTX and MYR+MTX groups. But, we can say that MYR treatment is more effective than CAS treatment for the reduction of MDA (see Figure 1). The amount of total GSH (nmol/g tissue) significantly decreased in the MTX group when compared to controls. Whereas, it increased in the CAS+MTX and MYR+MTX groups (see Figure 2). We can say that significant reduction of the SOD (U/g protein), GPX (U/g protein), and CAT (katal/g protein) enzyme activities were observed according to the control group in the MTX group but, CAS and MYR treatments were elevated and close to controls (see Figures 3, 4, and 5).

***Histopathological results***

**Figure 6:**
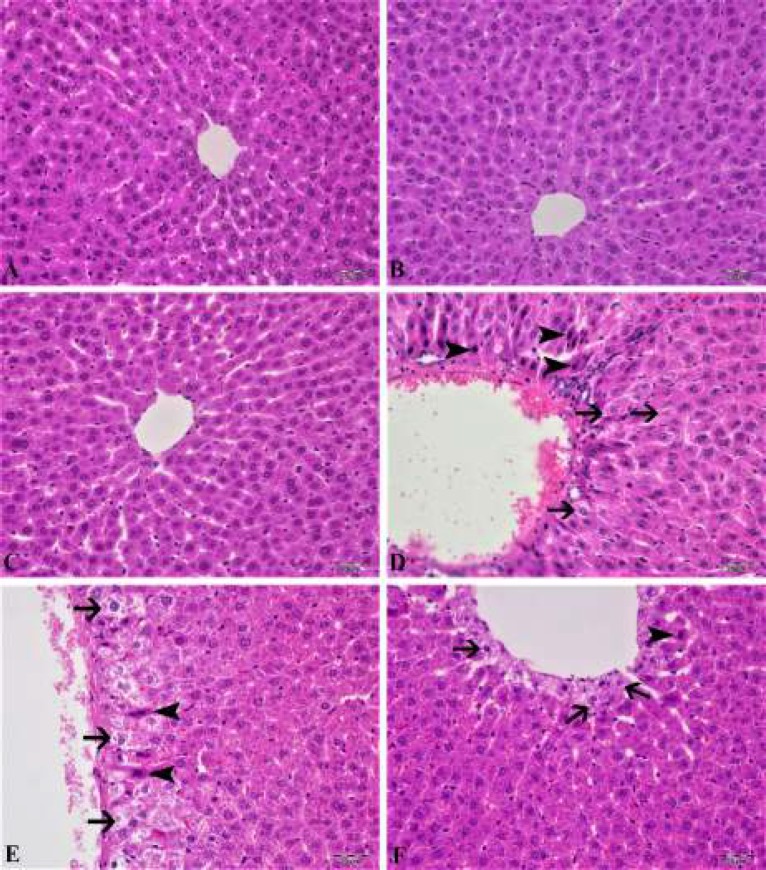
In control, CAS and MYR groups, hydropic degeneration (arrows) in hepatocyte, coagulation necrosis (arrowheads), and hyperemia in a vein (D) were normal histological appearance (A, B and C) in the liver tissue. In the CAS+methotrexate group: moderate hydropic degeneration (arrows) in hepatocytes, coagulation necrosis (arrowheads), mild hyperemia in veins (E). In the MYR+methotrexate group: mild hydropic degeneration (arrows) in hepatocytes, very little coagulation necrosis (arrowheads) (F), H & E, Bar: 20μm

Results of the histopathological study are presented in Figure 6 and Table 1. When the liver tissues of control, CAS, and MYR groups were examined, normal histological structure was seen (see Figures 6 A-B-C). When the liver tissues of the rats in the MTX group were examined, severe hydropic degeneration and coagulation necrosis in hepatocytes and severe hyperemia in veins were detected both in the parietal region and the portal region (see Figure 6D). CAS+MTX group showed moderate liver hydropic degeneration, especially in the periasiner area, moderate hyperemia in veins, and mild coagulation necrosis in sinusoids (see Figure 6E). There was a statistically significant difference compared to the MTX group (*P*<0.05). In liver tissues of the MYR+MTX group, hydropic degeneration in the periasiner region and very few hepatocyte coagulation necroses were observed (see Figure 6F). When compared with the MTX group, there was a statistically significant difference (*P<*0.05).

***Immunohistochemical results***

**Figure 7:**
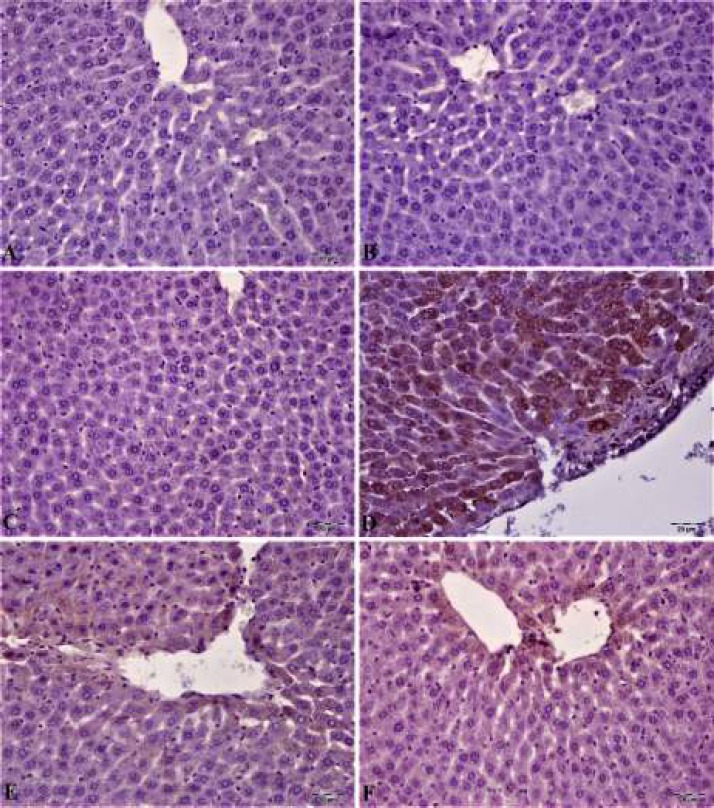
Caspase-3, an apoptosis mediator, expression of liver tissue was negative (A, B, and C) in control, CAS, and MYR groups. In the methotrexate group, severe caspase-3 expression (D) in hepatocytes. In the CAS+methotrexate group, moderate caspase-3 expression. In MYR+methotrexate group, caspase-3 expression was mild in hepatocytes, IP, 20 μm

In liver tissue immunohistochemical examination of control, CAS, and MYR groups no caspase-3 and 8-OHdG expressions were observed (see Figures 7/8 A-B-C). In the MTX group, caspase-3 and 8-OHdG expressions were seen at severe levels. There was a statistically significant difference when compared to these groups (*P<*0.05). Caspase-3 and 8-OHdG expression was mild in hepatocytes in the CAS+MTX group and Caspase-3 expression in the MYR+MTX group was less. Statistically significant (*P<*0.05) caspase-3 expression difference was seen in hepatocytes when these groups were compared with the MTX group. Histopathological and immunohistochemical findings were summarized in Table 1.

## Discussion

The mechanism of the hepatotoxic effect of MTX is a complex issue that is not yet fully explained. But, oxidative stress is mostly responsible for the unwanted effects of different chemotherapy drugs such as MTX and cisplatin. Also, oxidative stress is implicated as a side effect mechanism of MTX in various organs in previous studies (4, 25, 26). Drug toxicity studies have focused on oxidative stress. Oxidative stress generally results in lipid, protein, and DNA damage (27, 28). Increased lipid peroxidation has been shown in various hepatotoxicity studies. MTX is a folic acid antagonist that is widely used as an anti-inflammatory agent in inflammatory diseases such as psoriasis and dermatomyositis, also as a chemotherapeutic agent in some types of cancer. It has been determined in experimental animal studies that therapeutic effects have several side effects depending on the short and long-term usage of MTX. In this study, we aimed to investigate the possible effects of CAS and MYR on mitigating the hepatotoxic effect of MTX in experimental animals. For this purpose, we evaluated the biochemical parameters such as SOD and GPX activities, GSH and MDA levels, 8-hydroxy-2′-deoxyguanosine (8-OHdG) expression, and proapoptotic proteins such as caspase-3 expression levels in tissues. In the experimental studies, intraperitoneal administration of 20 mg/kg MTX in rats was found to be sufficient for hepatic and cholestatic type toxicity. In our study, we administered a single dose of 20 mg/kg intraperitoneally in rats, as described in the toxicity studies in the literature (29, 30). Also, in some studies (29, 30), rats were sacrificed on the fifth day after intraperitoneal MTX drug administration and found that hepatotoxicity was sufficient in this period (5^th^ day). 

**Figure 8:**
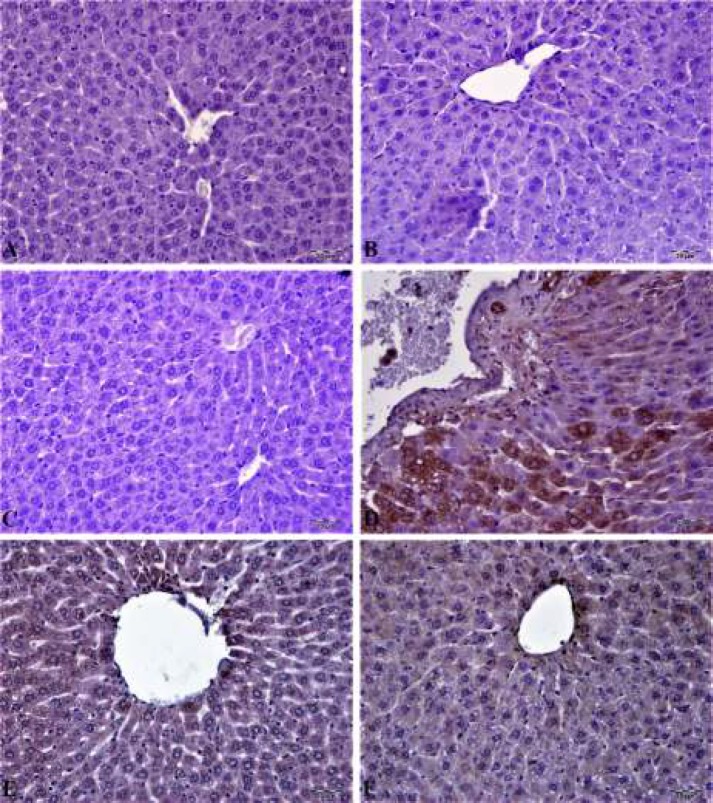
In control, CAS, and MYR groups, cytoplasmic 8-hydroxy-2′-deoxyguanosine (8-OHdG) expression was negative in liver tissues (A, B and C). In the methotrexate group, severe cytoplasmic 8-OHdG expression in hepatocytes (D). In the CAS+methotrexate group, moderate expression of 8-OHdG in hepatocytes in the periapical region and in the MYR+methotrexate group, mild expression of 8-OHdG in the hepatocytes in the pericentral region, IP, 20 μm

**Table 1:** Scores of Caspase-3, 8--hydroxy-2′-deoxyguanosine, hydropic degeneration, coagulation necrosis, and hyperemia in veins in the histopathological and immunohistochemical findings

	**Control** **group**	**Casticin group**	**Myricetin group**	**Methotrexate** **group**	**Casticin+** **Methotrexate** **group**	**Myricetin+ Methotrexate** **group**
Hydropic Degeneration	-	-	-	+++	++	+
Coagulation Necrosis	-	-	-	+++	+	+
Hyperemia in veins	-	-	-	+++	++	+
Caspase-3	-	-	-	+++	++	+
8--hydroxy-2′-deoxyguanosine	-	-	-	+++	++	+

The sections were evaluated as no (-), mild (+), moderate (++), and severe (+++) according to histopathological findings.

So far in many studies, oxidative stress induced by MTX is implicated in the liver, kidney, and testis toxicity (16, 29, 31). Increasing these oxidative stress indices may cause morphological and physiological changes in the liver tissue (32). Oxidative stress is explained as the degradation of the equilibrium state between the oxidant and antioxidant system in favor of the oxidant system. MDA is the most known marker for the level of lipid peroxidation (33-35). Important roles of SOD, GSH, GPx, and CAT within the antioxidant defense mechanism reduce or completely eliminate the hazardous effects of ROS (36, 37). Antioxidant enzyme system plays a primary role in the protection against oxidative stress and inflammation (38, 39). MDA and GSH levels, and SOD, GPx, and CAT activities were measured so that biochemical parameters could be revealed in liver toxicity caused by MTX. Previous research has indicated that MTX-induced hepatotoxicity is associated with consumption of liver antioxidant enzymes such as CAT, SOD, GPx, and GSH (30). GSH is one of the most considerable molecules in cellular defense against reactive oxygen species or nitrogen-based reactive compounds or oxidative stress. Reduced GSH is required for detoxification of xenobiotics (27, 40, 41). Decreased cellular GSH levels and capacity of GSH synthesis are sensitive to radiation and certain drugs. A study found that in the MTX group, glutathione (GSH) levels decreased, and malondialdehyde (MDA) levels, a marker of lipid peroxidation, increased in the liver, kidney, and small intestine (4). The increased lipid peroxidation may be attributed to alterations in the antioxidant defense system, including the enzyme glutathione peroxidase (GPx) as well as the reduced glutathione (GSH), which normally protects the biological system against free radical-induced toxicity (42). In the previous study, it was shown that TBARS levels and hepatic caspase-3 expression were significantly elevated, but TAC and GSH levels and CAT and SOD activities decreased in hepatic tissue. Additionally, down-regulation of hepatic caspase-3 expression via antioxidant treatment was shown (43). In our study, a significant increase in liver antioxidant enzymes SOD and GPx and CAT enzyme activities were detected in CAS+MTX and MYR+MTX groups compared to the MTX group (Figure 3, 4, 5). Also, the MDA level decreased in these treatment groups compared to the MTX group only (Figure 1). 

Oxidative stress and inflammation events are two principal mechanisms to cause apoptosis of liver cells (44). Many studies have proved the activation of proapoptotic proteins in liver toxic damage induced by MTX (45, 46). Apoptosis starts by multiple events that lead to the activation of a family of caspases or proteases. Caspases are responsible for the morphologic and biochemical characteristics of apoptotic cells (47). Caspases are significant signaling molecules of apoptosis, detection of caspases determinative for a primary phase in apoptosis mechanism (48). It was reported that caspase-3 is one of the most major proteases that start both the extrinsic and intrinsic apoptosis pathways and also a marker of the irreversible point of the apoptosis (43). MTX leads to mitochondrial swelling and membrane damage activating the caspase cascade (49). Caspase-3 is a frequently activated death protease, catalyzing the specific cleavage of many key cellular proteins. Overexpression of the pro-apoptotic mediator Caspase-3 in MTX-induced tissue damage was previously reported in different studies (9, 50, 51). Immunohistochemical examination in this study showed that liver toxic damage induced by MTX was attended by promotion of caspase-3 expression in hepatocytes. These data demonstrated a possible involvement of these mediators in induced by MTX liver damage. One of the most abundant oxidative DNA adducts is 8-hydroxy-2′-deoxyguanosine (8-OHdG) (52), which has been estimated to represent approximately 5% of all oxidative adducts (53). Among these damages, 8-OHdG is the predominant and most plenty oxidative product formed in nuclear and mitochondrial DNA. Although numerous DNA enzymatic repair and non-enzymatic antioxidant protect in organisms to sustain genomic stability, the unrepaired 8-OHdG in DNA can obtain in G:T transversion and further start carcinogenesis (54). It has been suggested that oxidative damage to DNA induced several diseases, involving cancer (55), aging (56), neurodegenerative diseases (57), and cardiovascular or infectious diseases (58). Therefore, 8-OHdG is not only a determiner of endogenous oxidative DNA damage but also useful for early diagnosis and evaluation of sensitive population. In our study, cytoplasmic 8-OHdG is the most damaging oxidative product in nuclear and mitochondrial DNA. Expression level was high in MTX administration group. But 8-OHdG expression level significantly reduced due to CAS and MYR treatments. 

One of the safest ways to determine MTX-induced liver toxicity is liver biopsy and histopathological examination. In this study, we also examined changes in liver tissue according to the histopathological scoring system. Semiquantitative scoring due to oxidative injury and apoptosis is an evaluation method used to demonstrate organ damage due to MTX in an experimental study (59). In our histopathological examination, the hydropic degeneration, hyperemia in veins in hepatocyte, coagulation necrosis expansion in sinusoids and hepatocyte are evaluated. It has been found that there are significant pathological changes due to single dose MTX administration in the liver, but the severity of pathological changes such as moderate hyperemia in veins, mild hydropic degeneration in the periasiner region and very few hepatocyte coagulation necroses in the liver due to CAS and MYR treatments are seen.

## Conclusion

In light of these results, it can be said that CAS and MYR have therapeutic effects on the liver, which is explained by free radical scavenger activity and alleviation of injury caused by ROS production, promote antioxidant defense by increasing antioxidant enzymes production or activity, are DNA protective and have the potential as antiapoptotic agents through reduction of apoptotic changes induced by MTX.
